# MAGE (Multimodal AI-Enhanced Gastrectomy Evaluation): Comparative Analysis of Machine Learning Models for Postoperative Complications in Central European Gastric Cancer Population

**DOI:** 10.3390/cancers18030443

**Published:** 2026-01-29

**Authors:** Wojciech Górski, Marcin Kubiak, Amir Nour Mohammadi, Maksymilian Podleśny, Gian Luca Baiocchi, Manuele Gaioni, S. Vincent Grasso, Andrew Gumbs, Timothy M. Pawlik, Bartłomiej Drop, Albert Chomątowski, Zuzanna Pelc, Katarzyna Sędłak, Michał Woś, Karol Rawicz-Pruszyński

**Affiliations:** 1 Department of Surgical Oncology, Medical University of Lublin, Radziwiłłowska 13 St., 20-080 Lublin, Polandzuzanna.pelc@umlub.edu.pl (Z.P.); katarzyna.sedlak@umlub.edu.pl (K.S.); 2 Department of Clinical and Experimental Sciences, Surgical Clinic, University of Brescia, and Third Division of General Surgery, Spedali Civili Di Brescia, 25123 Brescia, Italy; 3 Bartlett School of Architecture, University College of London, London WC1H 0QB, UK; 4 Department of Electrical and Computer Engineering, University of New Mexico, Albuquerque, NM 87106, USA; 5 Department of Medical Education and Scholarship, Rowan-Virtua School of Osteopathic Medicine, Rowan University, Stratford, NJ 08084, USA; 6 Hôpital Antoine Béclère, Assistance Publique-Hôpitaux de Paris, 157 Rue de la Porte de Trivaux, 92140 Clamart, France; 7 Department of General-, Visceral-, Vascular- and Transplantation Surgery, University of Magdeburg, 39120 Magdeburg, Germany; 8 Department of Surgery, The Ohio State University Wexner Medical Center and James Comprehensive Cancer Center, Columbus, OH 43210, USA; 9 Department of Computer Science and Medical Statistics with the e-Health Laboratory, Medical University of Lublin, 20-090 Lublin, Poland

**Keywords:** gastric cancer, multimodal treatment, machine-learning, postoperative complications

## Abstract

**Introduction**: By leveraging dedicated datasets and predictive modeling, machine-learning (ML) algorithms can estimate the probability of both short- and long-term outcomes after surgery. The aim of this study was to evaluate the ability of ML-based models to predict postoperative complications in patients with gastric cancer (GC) undergoing multimodal therapy. In particular, we aimed to develop a free, publicly accessible online calculator based on preoperative variables. **Materials and Methods**: Patients with histologically confirmed locally advanced (cT2-4N0-3M0) GC who underwent multimodal treatment with curative intent between 2013 and 2023 were included in the study. ML models evaluation pipeline was used with Stratified 5-Fold Cross-Validation. **Results**: A total of 368 patients were included in the final analytic cohort. Among five algorithm classes under 5-fold cross-validation, Compute Area Under the Receiver Operating Characteristic Curve (ROC AUC) was 0.9719, 0.9652, 0.9796, 0.8339 and 0.7581 for XGBoost, Catboost, Random Forest, SVM and Logistic Regression, respectively. Macro F1 was 0.8714, 0.5094, 0.8820, 0.8714 and 0.4579 for XGBoost, SVM, Random Forest, CatBoost and Logistic Regression, respectively. Overall Accuracy was 0.8897, 0.5980, 0.8885, 0.8750 and 0.5466 for XGBoost, SVM, Random Forest, CatBoost and Logistic Regression models, respectively. **Conclusions**: In this Central and Eastern European cohort of patients with locally advanced GC, ML models using non-linear decision rules-particularly Random Forest and XGBoost- substantially outperformed conventional linear approaches in predicting the severity of postoperative complications. Prospective external validation is needed to clarify the model’s clinical utility and its potential role in perioperative decision support.

## Simple Summary

Patients undergoing surgery for gastric cancer face a meaningful risk of postoperative complications, yet reliable tools to predict who is most at risk are still limited. In this study, we aimed to support preoperative decision-making by developing machine-learning models trained on data from gastric cancer patients receiving multimodal therapy at the Department of Surgical Oncology, Medical University of Lublin. If validated in future studies, this approach could help clinicians estimate surgical risk more accurately, individualize treatment planning, and improve perioperative safety. We also developed a free, user-friendly online risk calculator to facilitate clinical use.

## 1. Introduction

Despite major advances in multimodal therapy for gastric cancer (GC) over the past several decades, surgery remains the global standard of care for locally advanced disease [1]. However, both total and organ-preserving gastrectomy with D2 lymph node dissection are associated with substantial postoperative morbidity and mortality, with mortality rates reported as high as 20%, particularly in low-volume Western centers [2,3]. Perioperative complications, a major clinical concern following GC surgery, have been reported systematically in the European setting [4,5]. Evaluation of over 1300 gastrectomies revealed an overall complication incidence of 29.8%, with the most frequent complication being nonsurgical infections, anastomotic leak, abdominal fluid collections and pleural effusion (23%, 9.8%, 9.3% and 8.3%, respectively).

Because of disease heterogeneity and the complexity of the procedure, reliably predicting postoperative complications after GC surgery remains challenging. With the rapid integration of artificial intelligence (AI) into surgical oncology, technology-driven tools may help improve the quality and consistency of surgical care worldwide [6,7,8]. Machine learning (ML), one of the core subsets of AI, utilizes partial labeling of the data (supervised learning) or the structure detected in the data itself (unsupervised learning) to make clinical predictions [9]. Thus, by analyzing dedicated datasets and using predictive models, ML algorithms can accurately predict the likelihood of short- and long-term outcomes following surgery [10,11].

While several, including ML-driven, prediction models of postoperative complications after gastric cancer surgery have been recently proposed [12,13], most originate from Asian cohorts, whereas evidence from Central-Eastern Europe-where the incidence of GC remains comparatively high for the Western world-remains scarce. Therefore, the objective of the current study was to define the potential of ML-based models to predict postoperative complications among GC patients undergoing multimodal therapy. In particular, we sought to create a free accessible online calculator (Multimodal AI-enhanced Gastrectomy Evaluation, MAGE) utilizing preoperative variables.

## 2. Materials and Methods

### 2.1. Data Source, Study Design and Definitions

Individuals with locally advanced (cT2-4N0-3M0) adenocarcinoma who underwent multimodal treatment with curative intent between 2013 and 2023 in the Department of Surgical Oncology, Medical University of Lublin, Poland were included in the final analytic cohort. The initial date of patient recruitment was set due to the standardization of neoadjuvant chemotherapy (NAC) with 5-fluorouracil and platinum derivatives, reflecting current evidence-based clinical GC guidelines [1]. Patients who had not undergone resection, had early or metastatic GC at the time of initial diagnosis, had no postoperative complications (Clavien-Dindo Grade 0) who underwent palliative care, had died before the end of curative-intent planned treatment (Clavien-Dindo Grade V), or had incomplete clinical or pathological information were excluded. All patients provided informed consent for the proposed oncological treatment. In addition, as part of admission and care in a tertiary academic hospital, patients consented to the use of their medical data for research purposes in accordance with institutional policies and applicable regulations. The retrospective use of the clinical database for the development of a machine-learning prediction model was approved by the Institutional Review Board/Bioethics Committee (KE—0254/331/2024) and adhered to the guidelines of the Strengthening the Reporting of Observational Studies in Epidemiology (STROBE) [14] and TRIPOD-AI guidance for prediction modeling [15]. All procedures were performed in accordance with current revisions of the Declaration of Helsinki.

### 2.2. Perioperative Chemotherapy

Patients were scheduled for treatment with a combination of platinum- and fluoropyrimidine-based agents. The preferred regimen was FLOT-4, which consisted of docetaxel 50 mg/m^2^ on day 1, oxaliplatin 85 mg/m^2^ on day 1, leucovorin 200 mg/m^2^ on day 1, and 5-fluorouracil 2600 mg/m^2^ on day 1 of each cycle, repeated every 14 days [16]. Taking into account the inclusion period and the presence of comorbidities that precluded FLOT-4 administration, some patients received an EOX/ECF regimen (epirubicin 50 mg/m^2^ and oxaliplatin 130 mg/m^2^ on day 1, with capecitabine 625 mg/m^2^ administered twice daily on days 1–21, repeated every three weeks). After a 4–5-week interval, patients were referred for surgical treatment.

### 2.3. Gastrectomy and Lymphadenectomy

Gastrectomy consisted of total or subtotal radical resection and D2 lymphadenectomy, according to third and fourth edition of Japanese Gastric Cancer Association (JGCA) recommendations, followed by ex vivo seperation and division into specific separate stations containers for the further pathological examination.

### 2.4. Variables and Outcomes

Based on clinical relevance, 18 preoperative features used for model inputs and primary analysis included sex, age at surgery, weight, height, body mass index (BMI), Prognostic Nutritional Index (PNI) [17], baseline morphological parameters (hemoglobin, WBC, neutrophils, albumin, platelet count, CRP), AJCC [18] clinical tumor (cT) and nodal (cN) stages, Lauren histological subtype [19], Grading, receipt of NAC and number of NAC cycles. Postoperative complications were evaluated through Comprehensive Complication Index (CCI), with severe complications defined as >30), Clavien-Dindo Classification (CDC), with severe complications defined as >grade II) [20].

### 2.5. Statistical Analysis and Data Preprocessing

Variables with a normal distribution (*p* > 0.05) were reported as Mean ± SD, while non-normal variables (*p* < 0.05) were reported as Median [IQR]. The risk of serious postoperative complications was quantified using odds ratios (ORs) with 95% confidence intervals (CIs). Survival was compared with the log-rank test, and hazard ratios (HRs) with 95% CIs were estimated. Multivariable analyses were performed using logistic regression for complications and Cox proportional hazards models for survival. Variable selection in both multivariable models was based on backward elimination, and candidate predictors were drawn from variables that were significant in univariable analyses. A two-sided *p* value < 0.05 was considered statistically significant. In cases of missing values in the dataset, imputation was performed using the K-Nearest Neighbors (K-NN) method to maintain dataset completeness, with the parameter n_neighbors set to 5. The imputation process was carried out using the KNNImputer class, followed by rescaling for numerical columns to restore original values. Special attention was paid to categorical variables. For missing nominal data, a dedicated multiple imputation algorithm, Multiple Imputation by Chained Equations (MICE), with 20 iterations, was applied to preserve the correlation structure between variables. The categorical variable encoding process was expanded with target encoding for high-cardinality variables, while binary variables retained the traditional 0/1 approach. Analyses were conducted in Python 3.13 using Pandas, NumPy, SciPy, Scikit-learn, XGBoost, and CATBoost. To ensure clinical interpretability and preserve the original meaning of biological markers, the final models were trained on the full set of clinical features without dimensionality reduction. Model interpretability was subsequently addressed using SHAP analyses reported for the best-performing tree-based model.

### 2.6. Data Standardization, Machine Learning Models and Study Outcomes

Data standardization was performed using RobustScaler to provide greater robustness to outliers. To ensure clinical interpretability and preserve the original meaning of biological markers, the final models were trained on the full set of clinical features without dimensionality reduction. Model interpretability was subsequently addressed using SHAP analyses reported for the best-performing tree-based model. The study architecture involved the implementation and comparison of five ML model classes, specifically adapted for the multiclass classification problem with an imbalanced class distribution. Multinomial logistic regression was enhanced with advanced regularization techniques, implementing an elastic mix of L1 and L2 regularization (ElasticNet) with a mixing coefficient α = 0.5. Cost function optimization was performed using the Newton-CG algorithm with exact Hessian computation, which proved more efficient for medium-scale problems than the standard L-BFGS approach. The primary endpoint was estimation of classification metrics (macro-AUC, macro-F1, accuracy) under cross-validation for each ML model. To provide a clinical rationale for why a preoperative model that predicts postoperative complication severity may be useful for perioperative risk stratification and patient counseling, the secondary outcome was overall survival (OS), defined as the time elapsed between gastrectomy and death or last follow-up. Kaplan–Meier curves were generated for OS with group stratifications by CDC grade. Flowchart of the study is shown in Supplementary Figure S1.

## 3. Results

A total of 368 patients who underwent curative-intent multimodal treatment for locally advanced GC were included in the final analytic cohort. Most patients were male (n = 222, 59.5%) and the median age at the time of surgery was 63. A substantial subset of individuals were overweight (n = 148, 40.22%) and had intestinal histological subtype (n = 174, 46.6%). Among the entire cohort, 146 (39.6%) patients had Grade I complications, 111 (30.1%) individuals had Grade II complications, while 57 (15.49%) and 54 (14.67%) patients required surgical, endoscopic or radiological intervention, or intensive care unit management (Grade III and IV, respectively) (Table 1).

### 3.1. Machine Learning Models Performance–Cross Validation

Among five algorithm classes under 5-fold cross-validation, Compute Area Under the Receiver Operating Characteristic Curve (ROC AUC) was 0.9719, 0.9652, 0.9796, 0.8339 and 0.7581 for XGBoost, Catboost, Random Forest, SVM and Logistic Regression, respectively. Macro F1 was 0.8714, 0.5094, 0.8820, 0.8714 and 0.4579 for XGBoost, SVM, Random Forest, CatBoost and Logistic Regression, respectively. Overall Accuracy was 0.8897, 0.5980, 0.8885, 0.8750 and 0.5466 for XGBoost, SVM, Random Forest, CatBoost and Logistic Regression models, respectively (Table 2).

### 3.2. Machine Learning Models Performance–Classification Report

*Grade I*

For XGBoost ML model, precision, recall, F1-score and support for Grade I complications were 0.82, 0.80, 0.81 and 88, respectively. For Random Forest ML model, precision, recall, F1-score and support for Grade I complications were 0.74, 0.77, 0.76 and 88, respectively. For CatBoost ML model, precision, recall, F1-score and support for Grade I complications were 0.77, 0.75, 0.76 and 88, respectively. Of note, for Linear Regression model, precision, recall, F1-score and support for Grade I complications were 0.49, 0.51, 0.50 and 88, respectively.

*Grade II*

For the XGBoost ML model, precision, recall, F1-score and support for Grade II complications were 0.81, 0.90, 0.85 and 88, respectively. For the Random Forest ML model, precision, recall, F1-score and support for Grade II complications were 0.83, 0.89, 0.86 and 88, respectively. For the CatBoost ML model, precision, recall, F1-score and support for Grade II complications were 0.81, 0.81, 0.81 and 88, respectively. Of note, for Linear Regression model, precision, recall, F1-score and support for Grade II were 0.53, 0.27, 0.36 and 88, respectively.

*Grade III*

For the XGBoost ML model, precision, recall, F1-score and support for Grade III complications were 0.87, 0.73, 0.79 and 37, respectively. For the Random Forest ML model, precision, recall, F1-score and support for Grade III complications were 0.93, 0.73, 0.82 and 37, respectively. For the CatBoost ML model, precision, recall, F1-score and support for Grade III complications were 0.89, 0.84, 0.86 and 37, respectively. Of note, for Linear Regression model, precision, recall, F1-score and support for Grade III were 0.15, 0.16, 0.16 and 37, respectively.

*Grade IV*

**Figure 1:**
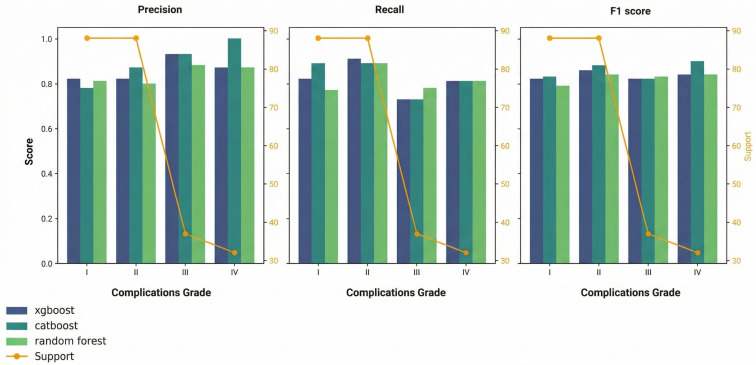
Comparison of selected ML model performance for predicting postoperative complications.

For the XGBoost ML model, precision, recall, F1-score and support for Grade IV complications were 0.88, 0.88, 0.88 and 32, respectively. For the Random Forest ML model, precision, recall, F1-score and support for Grade IV complications were 0.87, 0.81, 0.84 and 32, respectively. For the CatBoost ML model, precision, recall, F1-score and support for Grade IV complications were 0.78, 0.88, 0.82 and 32, respectively. Of note, for Linear Regression model, precision, recall, F1-score and support for Grade IV were 0.25, 0.53, 0.34 and 32, respectively. Comparison of selected ML model performance for predicting postoperative complications are shown in Figure 1.

### 3.3. Confusion Matrix Analysis

*XBoost*

The confusion matrix analysis for the training set XGBoost model demonstrated most classification errors in Grade I cases being mistaken for Grade II (n = 12). For Grade III, the main errors were due to misclassification as Grade I (n = 6) and Grade II (n = 4). The ROC AUC values for Grades I, II, III and IV were 0.95, 0.98, 0.95 and 0.98, respectively. Among the most important features determining postoperative complications severity were clinical lymph node status (cN; 0.1146), NAC receipt (0.0939), and Prognostic Nutritional Index (PNI; 0.0706).

*Random Forest*

The confusion matrix analysis for the training set Random Forest model demonstrated most mutual misclassifications of Grade I and II (n = 8 and n = 10, respectively). For Grade III, the main errors were due to misclassification as Grade I (n = 8). The ROC AUC values for Grades I, II, III and IV were 0.96, 0.97, 0.95 and 0.93, respectively. Among the most important features determining the postoperative complications severity were Albumin, Neutrophil and CRP levels (0.0796, 0.0795 and 0.0795, respectively), followed by age at surgery (0.0718) and Protein level (0.0687).

*CatBoost*

The confusion matrix analysis for the training set CatBoost model had the most mutual missclassifications of Grade I and II (n = 14 and n = 8, respectively). For Grade III, the main errors were due to misclassification as Grade I and II (both n = 4). The ROC AUC values for Grades I, II, III and IV were 0.96, 0.97, 0.96 and 0.96, respectively. Similarly to Random Forst model, among the most important features determining the postoperative complications severity were Neutrophil and CRP levels (0.0796 and 0.0795, respectively), followed by WBC (0.0719) and NAC receipt (0.0708). To enhance interpretability of the non-linear models, Shapley Additive Explanations (SHAP) are shown as Supplementary Figures to quantify the contribution of each preoperative feature to the predicted postoperative complication grade.

### 3.4. Survival Analysis

**Figure 2:**
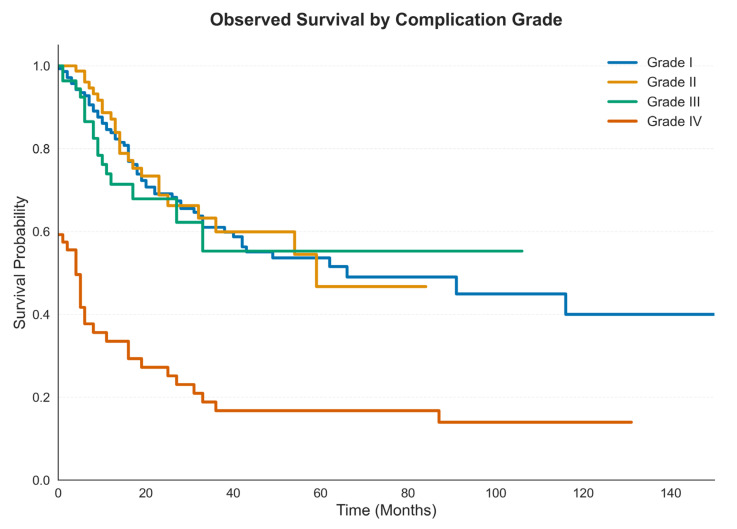
Kaplan Meier curves of OS estimates in locally advanced GC by type of postoperative complications.

Median OS in the overall cohort was 58 months. For patients with Grade I, II, III and IV complications median OS was 62 months, (95% CI: 62.0–81.0), 59 months (95% CI: 32.00–82.00), 57 months (95% CI: 27.00–88.00) and 4 months (95% CI: 0.0–8.0), respectively (Figure 2).

### 3.5. Model Interpretability and Feature Importance

**Figure 3:**
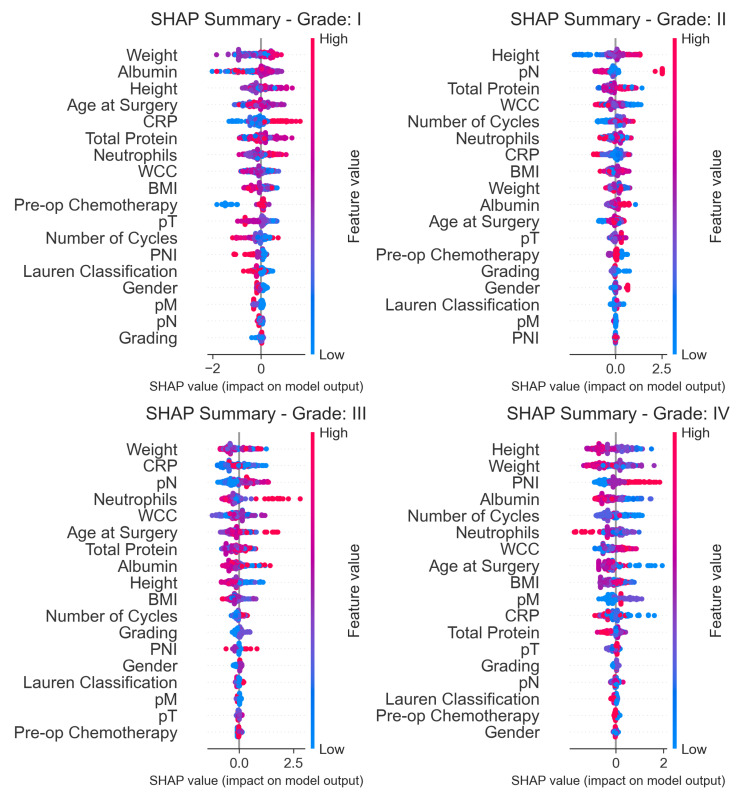
Global Feature Importance for the XGBoost model. The bar chart illustrates the top predictors of postoperative complications ranked by their relative importance score. The model prioritizes oncological factors, with pN stage and Preoperative Chemotherapy having the highest impact on prediction, followed by Prognostic Nutritional Index (PNI) and pT stage. Anthropometric and nutritional indicators (Height, Albumin, Weight) also play a significant role, outranking demographic variables like Age and Gender.

To validate the clinical relevance of the XGBoost model, we analyzed the global feature importance, which ranks variables based on their contribution to the model’s predictive accuracy. As presented in Figure 3, the model identified a combination of pathological, treatment-related, and anthropometric factors as the primary drivers of postoperative complications.

The analysis revealed that pathological lymph node staging (pN) was the single most critical predictor (importance score: 0.1146), followed by Pre-operative Chemotherapy (0.0939) and Perineural Invasion (PNI) (0.0706). This hierarchy suggests that the extent of oncological burden and the administration of neoadjuvant treatment are the dominant determinants of surgical morbidity in this cohort.

Furthermore, the model placed significant weight on patient-specific physiological markers. Height (0.0607), Albumin levels (0.0575), and Weight (0.0570) ranked within the top seven features, highlighting the role of nutritional status and body composition in risk stratification. Interestingly, standard demographic factors such as Gender (0.0514) and Age (0.0477) were found to be less influential than tumor biology (pT, pN, PNI) and nutritional markers.

## 4. Discussion

A recent European multicenter study reported lower rate of perioperative complications among patients undergoing minimally invasive subtotal gastrectomy compared with individuals after open subtotal gastrectomy, with no differences in outcomes after total gastrectomy, regardless of surgical approach [21]. These results, provided by high-volume referral centers, indicate further training and evaluation to support safe and effective integration of minimally-invasive techniques in multimodal treatment of GC. Moreover, marked differences between Western and Eastern populations have been reported in GC, including histologic subtype distribution (intestinal vs. diffuse), tumor location (distal vs. proximal), environmental exposures, dietary patterns, and the overall diagnostic workup. The higher incidence of gastric adenocarcinoma in Eastern countries has supported the implementation of screening programs, which in turn results in earlier-stage presentation. Surgical strategies also differ, as extended lymphadenectomy is routinely performed in many Asian centers. Lastly, systemic treatment approaches and preferred regimens vary substantially between the two regions [22]. Therefore, to the best of our knowledge, this is the first study from the West that evaluated utilization of ML to predict postoperative complications after gastrectomy. In comparative analyses, XGBoost model achieved the highest discrimination (macro-AUC: 0.95 and per-class AUC 0.98 for Grades II and IV) with consistently favorable macro-metrics. Of note, Random Forest model demonstrated perfect precision for Grade IV (1.00) and focused on nutritional and biochemical variables (albumin, neutrophils, CRP), indicating the clinical association between poor baseline status and possible postoperative complications. CatBoost model provided the most balanced performance across classes, with a prominent contribution from inflammatory markers. Misclassification patterns were similar across models and occurred most frequently between non-severe complications, Grades I and II. These findings suggest that separability might be improved by incorporating composite indices integrating inflammatory and nutritional status, followed by non-linear feature characteristics evaluation.

A systematic-review and meta-analysis of 38 studies compared the performance of ML and logistic regression (LR) models to predict postoperative outcomes for patients undergoing gastrointestinal surgery [23]. The authors reported a significant improvement in AUC when using ML over LR algorithms, which aligns with findings of the current study. While LR assumes a linear relationship between the independent variables and the log odds of the dependent variable, ML models can process substantial amounts of data and high dimensionality, allowing analysis of complex nonlinear relationships. Similarly to our study, findings of a scoping review investigating ML to predict postoperative complications after digestive surgery demonstrated good performance in integrating demographics, medical history, laboratory tests, and surgical details to predict severe complications after gastrectomy, with an AUC of 0.90 [24,25].

Given the clinical importance of the association between perioperative complications and favourable oncological outcomes after GC surgery [26], structured and standardized CDC proved to be a useful tool for risk stratification in the ML setting. A four-level grading allowed for more precise algorithm creation when compared with traditional binary approach. Moreover, application of advanced ML techniques, such as as data imputation (K-NN, MICE), scaling (RobustScaler), and dimensionality reduction (PCA), can improve prediction quality further.

Evaluation of ML-driven models for time-sequential prediction of postoperative complications after gastrectomy among 4139 patients allowed improved accuracy (AUC 0.733) in detection of severe complications (Grade > 2) in 27.4% cases. Of note, only 7% of the cohort received NAC [13].

An example of innovative integration of nutritional and inflammatory markers within a ML framework, offering a novel approach to survival estimation in advanced GC was recently reported in a Polish study by Matysiak et al. [27]. Among 410 stage IV GC patients undergoing home parenteral nutrition (HPN). A Random Survival Forest (RSF) demonstrated strong predictive accuracy (C-index: 0.985–0.986) and effectively stratified patients by survival risk. Considering the importance of baseline characteristics (PNI, CRP, WBC) to determine possible postoperative complications after GC surgery, the current study demonstrated substantial differences in OS among different CDC grades.

Certain limitations of the current study need to be addressed. First, the retrospective design, limited sample size and lack of external validation may limit the generalizability of our findings. Secondly, class imbalances (e.g., a small number of Grade IV individuals) might have affected the sensitivity of the models in detecting rare but critical complications. Future prospective studies should consider using oversampling techniques (e.g., SMOTE) or weighted loss functions to improve the detection of high-risk patients [28]. Importantly, the “black-box” nature of many ML models can limit interpretability and undermine clinician confidence, particularly in health systems where AI adoption is emerging, including Poland. Enhancing transparency with explainable-AI techniques is therefore critical to facilitate clinical adoption among GC caregivers. While dimensionality reduction techniques can reduce collinearity, they limit interpretability because principal components do not correspond to directly actionable clinical variables. This limitation is particularly relevant in clinical risk prediction, where transparency is essential for trust and implementation. Therefore, we avoided dimensionality reduction and trained the models on the original clinical variables. To address potential multicollinearity among laboratory markers, we relied on the inherent feature selection capabilities of tree-based ensemble models (Random Forest, XGBoost) and utilized explainable-AI methods (SHAP) to provide feature-level explanations in clinically interpretable terms. Despite aiming to predict Clavien–Dindo Grades, the model was effectively trained only on Grades I–IV. Predictions for Grades 0 and V were therefore structurally impossible Given the cohort size and the multiclass endpoint, model overfitting is another potential limitation. We mitigated this risk by using stratified 5-fold cross-validation and reporting mean ± SD performance across folds, and by applying regularization and complexity-control strategies (including ElasticNet regularization for logistic regression and early stopping/validation-based tuning for boosting models). Nevertheless, the observed high discrimination-particularly for tree-based methods- may partially capture cohort-specific patterns. Despite these limitations, MAGE study demonstrates the feasibility of using ML methods to grade postoperative complications after multimodal GC treatment. A prospective study is planned to provide external validation of predictive performance and calibration, and to evaluate model clinical utility in routine workflow.

## 5. Conclusions

In this Central and Eastern European cohort of patients with locally advanced GC, ML models based on non-linear decision rules-particularly Random Forest and XGBoost-substantially outperformed conventional linear methods in predicting the severity of postoperative complications. While multinomial logistic regression showed only moderate discrimination (macro-AUC ~0.76), tree-based algorithms achieved excellent performance (macro-AUC ~0.97–0.98) with high overall accuracy. This suggests that postoperative morbidity risk is shaped by complex, non-linear interactions among routinely available preoperative variables. Clinically, the most influential predictors across models aligned with real-world risk biology and treatment pathways (e.g., nodal status, neoadjuvant chemotherapy, and nutritional/inflammatory markers such as PNI, CRP- and leukocyte-based indices), supporting the plausibility of the learned patterns. Taken together, these findings indicate that ML-based tools may facilitate personalized risk stratification, improve perioperative counseling, and enable more targeted prehabilitation and optimization prior to gastrectomy. However, given the retrospective single-center design and the class imbalance for higher-grade complications, prospective external validation is necessary before routine clinical implementation. The publicly accessible MAGE online calculator (http://servturbo2.synology.me:15012/, accessed on 12 January 2026) provides a practical platform for future multicenter validation and iterative model refinement.

## Figures and Tables

**Table 1:** Patient characteristics.

Variable	Total	Grade I	Grade II	Grade III	Grade IV	*p*-Value
		62.0	63.0	65.0	63.5	
Age at Surgery	63.0 (55.0–70.0)	(55.0–68.0)	(55.0–70.0)	(56.0–73.0)	(51.5–70.0)	0.278
Sex						
Female	149 (39.9%)	61 (41.8%)	41 (36.3%)	23 (38.3%)	24 (44.4%)	0.769
Male	222 (59.5%)	85 (58.2%)	70 (61.9%)	37 (61.7%)	30 (55.6%)	
Weight	76.1 ± 17.3	76.8 ± 17.8	79.3 ± 15.8	61.5 ± 21.8	67.2 ± 3.7	0.034
Height	169.1 ± 9.9	169.5 ± 9.2	171.4 ± 9.7	160.0 ± 13.0	165.0 ± 4.7	0.081
BMI	26.6 ± 3.9	25.2 ± 6.2	26.9 ± 3.3	27.9 ± 8.5	25.2 ± 2.0	0.904
CRP	1.3 [1.0–3.3]	2.2 [1.2–6.5]	1.1 [0.9–2.2]	1.0 [1.0–1.0]	1.0 [0.8–1.4]	0.280
Albumin	4.4 [4.2–4.6]	4.3 [4.2–4.5]	4.4 [4.2–4.6]	4.6 [4.4–4.8]	4.2 [4.1–4.2]	0.337
WBC	6.5 [5.5–8.4]	7.3 [5.5–8.5]	6.7 [5.6–8.2]	7.2 [6.9–7.6]	6.2 [5.2–6.3]	0.652
Neutrophils	4.5 [3.3–5.4]	4.7 [3.4–6.0]	4.7 [3.0–5.1]	4.1 [3.7–4.4]	4.4 [4.4–4.4]	0.910
Total Protein	6.7 ± 0.5	6.7 ± 0.6	6.8 ± 0.5	6.9 ± 0.5	6.6 ± 0.3	0.932
NAC cycles	4.0 [3.0–4.0]	4.0 [3.0–4.0]	4.0 [4.0–4.0]	4.0 [3.0–4.0]	3.0 [3.0–4.0]	0.001
ypT						0.543
T0	26 (7.0%)	12 (8.2%)	9 (8.0%)	3 (5.0%)	2 (3.7%)	
T1	36 (9.7%)	18 (12.3%)	11 (9.7%)	3 (5.0%)	4 (7.4%)	
T2	61 (16.4%)	22 (15.1%)	24 (21.2%)	8 (13.3%)	7 (13.0%)	
T3	157 (42.1%)	64 (43.8%)	41 (36.3%)	29 (48.3%)	23 (42.6%)	
T4	86 (23.1%)	26 (17.8%)	26 (23.0%)	16 (26.7%)	18 (33.3%)	
ypN						0.0107
N0	180 (48.9%)	72 (49.7%)	60 (54.5%)	25 (41.7%)	23 (44.2%)	
N1	48 (13%)	23 (15.9%)	15 (13.6%)	4 (6.7%)	6 (11.5%)	
N2	56 (15.2%)	23 (15.9%)	18 (16.4%)	8 (13.3%)	7 (13.5%)	
N3	84 (22.9%)	27 (18.5%)	17 (15.6%)	23 (38.3%)	16 (30.8%)	
Grading						0.254
G1	18 (4.8%)	7 (4.8%)	8 (7.1%)	2 (3.3%)	1 (1.9%)	
G2	154 (41.3%)	68 (46.6%)	44 (38.9%)	23 (38.3%)	19 (35.2%)	
G3	197 (52.8%)	70 (47.9%)	58 (51.3%)	35 (58.3%)	34 (63.0%)	
Lauren						0.150
Intestinal	174 (46.6%)	74 (52.1%)	50 (48.2%)	28 (46.7%)	22 (41.2%)	
Mixed	67 (18%)	26 (18.8%)	16 (16.2%)	9 (15.0%)	16 (30%)	
Diffuse	117 (31.4%)	41 (29.1%)	38 (35.6%)	23 (38.3%)	15 (28.8%)	

Continuous variables are presented as Mean ± SD (for normal distribution) or Median [IQR] (for non-normal distribution). *p*-values were calculated using the Shapiro–Wilk test for normality, followed by Kruskal–Wallis or ANOVA, and Chi-square test for categorical variables; BMI: Body Mass Index; CRP: C-reactive protein; WBC: White Blood Cell count; NAC cycles: Neoadjuvant Chemotherapy number of cycles; ypT–post pathological tumor stage; ypN–post pathological nodal stage.

**Table 2:** Cross-validation results of ML models.

Model	Accuracy (Mean ± SD)	Macro F1 (Mean ± SD)	Macro AUC (Mean ± SD)
Logistic Regression	0.5466 ± 0.0419	0.4579 ± 0.0433	0.7581 ± 0.0266
Random Forest	0.8885 ± 0.0249	0.8820 ± 0.0238	0.9796 ± 0.0076
SVM	0.5980 ± 0.0429	0.5094 ± 0.0275	0.8339 ± 0.0079
XGBoost	0.8897 ± 0.0189	0.8785 ± 0.0226	0.9719 ± 0.0161
CatBoost	0.8750 ± 0.0363	0.8714 ± 0.0349	0.9652 ± 0.0202

## Data Availability

Data processed in the current study can be shared upon reasonable request.

## Supplementary Materials

The following supporting information can be downloaded at: https://www.mdpi.com/article/10.3390/cancers18030443/s1, Figure S1: Study Flowchart; Figure S2: Confusion Matrixes (a–e); Figure S3: Feature Importance (a–e); Figure S4: SHAP (a,b).

## References

[1] Lordick F., Carneiro F., Cascinu S., Fleitas T., Haustermans K., Piessen G., Vogel A., Smyth E.C., ESMO Guidelines Committee (2022). Gastric cancer: ESMO Clinical Practice Guideline for diagnosis, treatment and follow-up. Ann. Oncol..

[2] Visser M.R., Voeten D.M., Gisbertz S.S., Ruurda J.P., van Berge Henegouwen M.I., van Hillegersberg R., Dutch Upper Gastrointestinal Cancer Audit (DUCA) Group (2024). Outcomes after gastrectomy according to the Gastrectomy Complications Consensus Group (GCCG) in the Dutch Upper GI Cancer Audit (DUCA). Gastric Cancer.

[3] Papenfuss W.A., Kukar M., Oxenberg J., Attwood K., Nurkin S., Malhotra U., Wilkinson N.W. (2014). Morbidity and mortality associated with gastrectomy for gastric cancer. Ann. Surg. Oncol..

[4] Baiocchi G.L., Giacopuzzi S., Marrelli D., Reim D., Piessen G., Matos da Costa P., Reynolds J.V., Meyer H.J., Morgagni P., Gockel I. (2019). International consensus on a complications list after gastrectomy for cancer. Gastric Cancer.

[5] Baiocchi G.L., Giacopuzzi S., Reim D., Piessen G., Costa P.M.D., Reynolds J.V., Meyer H.J., Morgagni P., Gockel I., Santos L.L. (2020). Incidence and Grading of Complications After Gastrectomy for Cancer Using the GASTRODATA Registry: A European Retrospective Observational Study. Ann. Surg..

[6] Hashimoto D.A., Rosman G., Rus D., Meireles O.R. (2018). Artificial Intelligence in Surgery: Promises and Perils. Ann. Surg..

[7] Fahim K., Narmin Z., Goykhman Y., Nir M., Amedeo C., Nir L., Aviad G., Eviatar N. (2025). Clinical outcomes, learning effectiveness, and patient-safety implications of AI-assisted HPB surgery for trainees: A systematic review and multiple meta-analyses. Artif. Intell. Surg..

[8] Yutaka E., Laura A., Giovanni C., Odysseas P.C., Timothy M.P. (2024). Application of artificial intelligence to hepatobiliary cancer clinical outcomes research. Artif. Intell. Surg..

[9] Gianluca R., Francesca P., Bianca P., Giusy P., Gianluca B., Micaela C., Roberto M., Roberto T. (2025). Role of artificial intelligence in the detection, assessment and outcome of gastroesophageal varices. Artif. Intell. Surg..

[10] Satapathy P., Pradhan K.B., Rustagi S., Suresh V., Al-Qaim Z.H., Padhi B.K., Sah R. (2023). Application of machine learning in surgery research: Current uses and future directions—Editorial. Int. J. Surg..

[11] Martin W., André S., Michael H.-K., Pascal P., Johanna M.B., Eva K., Ali M., Ali R., Rosa K., Felix N. (2022). Artificial intelligence for decision support in surgical oncology—A systematic review. Artif. Intell. Surg..

[12] Kunisaki C., Miyata H., Konno H., Saze Z., Hirahara N., Kikuchi H., Wakabayashi G., Gotoh M., Mori M. (2017). Modeling preoperative risk factors for potentially lethal morbidities using a nationwide Japanese web-based database of patients undergoing distal gastrectomy for gastric cancer. Gastric Cancer.

[13] Ri M., Nunobe S., Narita T., Seto Y., Kawazoe Y., Ohe K., Azuma L., Takeshita N. (2025). Time-sequential prediction of postoperative complications after gastric cancer surgery using machine learning: A multicenter cohort study. Gastric Cancer.

[14] von Elm E., Altman D.G., Egger M., Pocock S.J., Gotzsche P.C., Vandenbroucke J.P., Initiative S. (2007). Strengthening the Reporting of Observational Studies in Epidemiology (STROBE) statement: Guidelines for reporting observational studies. BMJ.

[15] Collins G.S., Moons K.G.M., Dhiman P., Riley R.D., Beam A.L., Van Calster B., Ghassemi M., Liu X., Reitsma J.B., van Smeden M. (2024). TRIPOD+AI statement: Updated guidance for reporting clinical prediction models that use regression or machine learning methods. BMJ.

[16] Al-Batran S.-E., Hofheinz R.D., Pauligk C., Kopp H.-G., Haag G.M., Luley K.B., Meiler J., Homann N., Lorenzen S., Schmalenberg H. (2016). Histopathological regression after neoadjuvant docetaxel, oxaliplatin, fluorouracil, and leucovorin versus epirubicin, cisplatin, and fluorouracil or capecitabine in patients with resectable gastric or gastro-oesophageal junction adenocarcinoma (FLOT4-AIO): Results from the phase 2 part of a multicentre, open-label, randomised phase 2/3 trial. Lancet Oncol..

[17] Kakiuchi Y., Kuroda S., Choda Y., Otsuka S., Ueyama S., Tanaka N., Muraoka A., Hato S., Kamikawa Y., Fujiwara T. (2023). Prognostic nutritional index is a prognostic factor for patients with gastric cancer and esophagogastric junction cancer undergoing proximal gastrectomy with esophagogastrostomy by the double-flap technique: A secondary analysis of the rD-FLAP study. Surg. Oncol..

[18] Mranda G.M., Xue Y., Zhou X.G., Yu W., Wei T., Xiang Z.P., Liu J.J., Ding Y.L. (2022). Revisiting the 8th AJCC system for gastric cancer: A review on validations, nomograms, lymph nodes impact, and proposed modifications. Ann. Med. Surg..

[19] Lauren P. (1965). The Two Histological Main Types of Gastric Carcinoma: Diffuse and So-Called Intestinal-Type Carcinoma. An Attempt at a Histo-Clinical Classification. Acta Pathol. Microbiol. Scand..

[20] Clavien P.A., Vetter D., Staiger R.D., Slankamenac K., Mehra T., Graf R., Puhan M.A. (2017). The Comprehensive Complication Index (CCI(R)): Added Value and Clinical Perspectives 3 Years “Down the Line”. Ann. Surg..

[21] Bencivenga M., Keywani K., Torroni L., Filippini F., Giacopuzzi S., Verlato G., Hoelscher A., D’Ugo D., Piessen G., Wijnhoven B. (2025). Perioperative Outcomes in Open Versus Minimally Invasive Gastrectomy For Gastric Cancer: A European Multicenter Study Based on the GASTRODATA Registry. Ann. Surg..

[22] Bickenbach K., Strong V.E. (2012). Comparisons of Gastric Cancer Treatments: East vs. West. J. Gastric Cancer.

[23] Wang J., Tozzi F., Ashraf Ganjouei A., Romero-Hernandez F., Feng J., Calthorpe L., Castro M., Davis G., Withers J., Zhou C. (2024). Machine learning improves prediction of postoperative outcomes after gastrointestinal surgery: A systematic review and meta-analysis. J. Gastrointest. Surg..

[24] Ravenel M., Joliat G.R., Demartines N., Uldry E., Melloul E., Labgaa I. (2023). Machine learning to predict postoperative complications after digestive surgery: A scoping review. Br. J. Surg..

[25] Shao S., Liu L., Zhao Y., Mu L., Lu Q., Qin J. (2021). Application of Machine Learning for Predicting Anastomotic Leakage in Patients with Gastric Adenocarcinoma Who Received Total or Proximal Gastrectomy. J. Pers. Med..

[26] Sedlak K., Rawicz-Pruszynski K., Mlak R., Van Sandick J., Gisbertz S., Pera M., Dal Cero M., Baiocchi G.L., Celotti A., Morgagni P. (2023). Textbook Oncological Outcome in European GASTRODATA. Ann. Surg..

[27] Matysiak K., Hojdis A., Szewczuk M. (2025). Survival Modelling Using Machine Learning and Immune-Nutritional Profiles in Advanced Gastric Cancer on Home Parenteral Nutrition. Nutrients.

[28] Xu Q., Ma L., Streuer A., Altrock E., Schmitt N., Rapp F., Klar A., Nowak V., Oblander J., Weimer N. (2025). Machine learning-based in-silico analysis identifies signatures of lysyl oxidases for prognostic and therapeutic response prediction in cancer. Cell Commun. Signal.

