# Supplementary material for: MAGE (Multimodal AI-Enhanced Gastrectomy Evaluation): Comparative Analysis of Machine Learning Models for Postoperative Complications in Central European Gastric Cancer Population
Source: Cancers (Basel). 2026 Jan 29;18(3):443. doi: 10.3390/cancers18030443 (PMC12896461; doi:10.3390/cancers18030443)
Supplement: Supplementary file 1 [file cancers-18-00443-s001.zip › cancers-4076988-supplementary.pdf]

MAGE (Multimodal AI-Enhanced Gastrectomy Evaluation): Comparative Analysis of Machine Learning Models for Postoperative Complications in Central European Gastric Cancer Population

Supplementary Figure S1. Study Flowchart

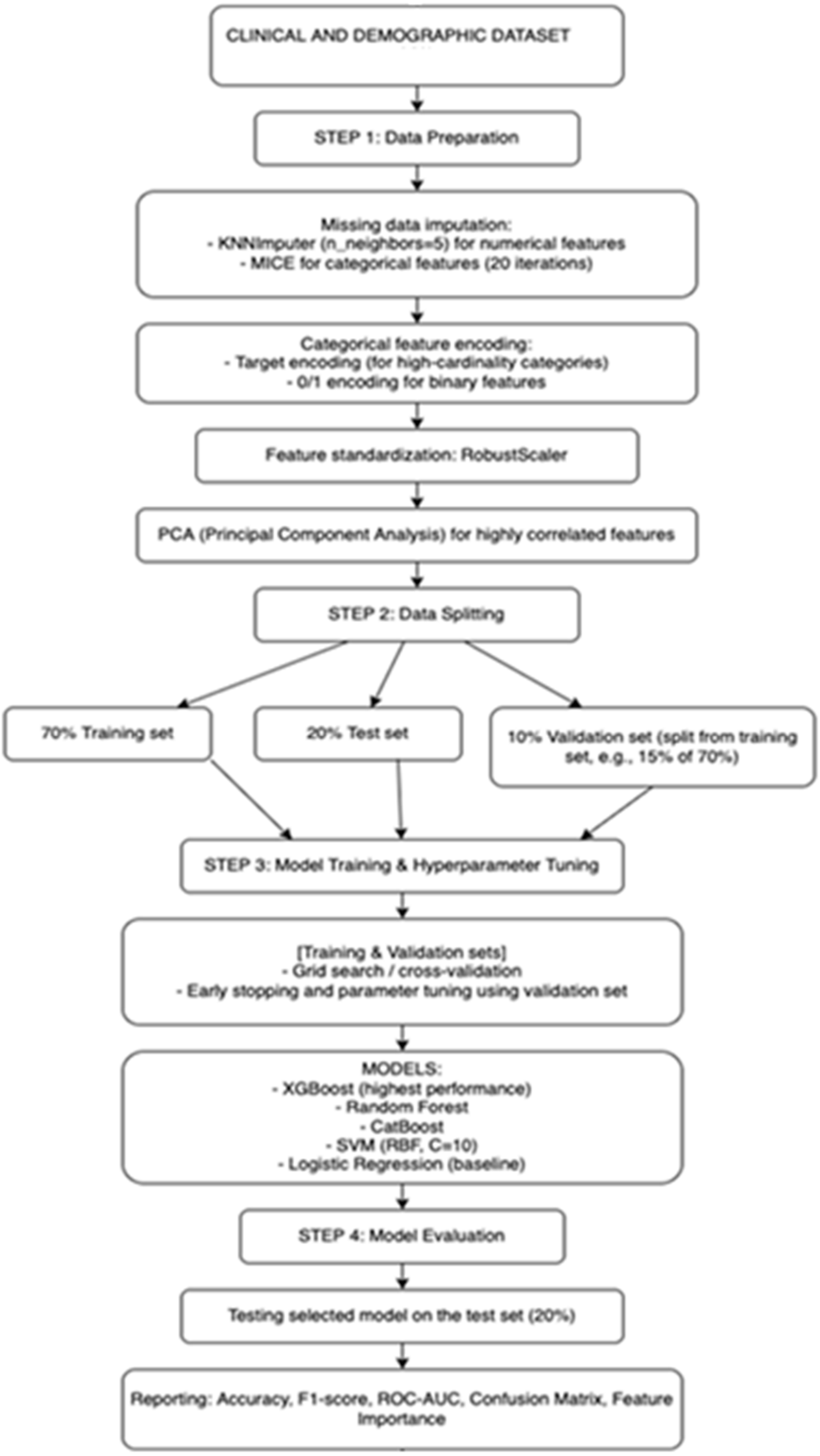

Supplementary Figure S2. Confusion Matrix

a) CatBoost

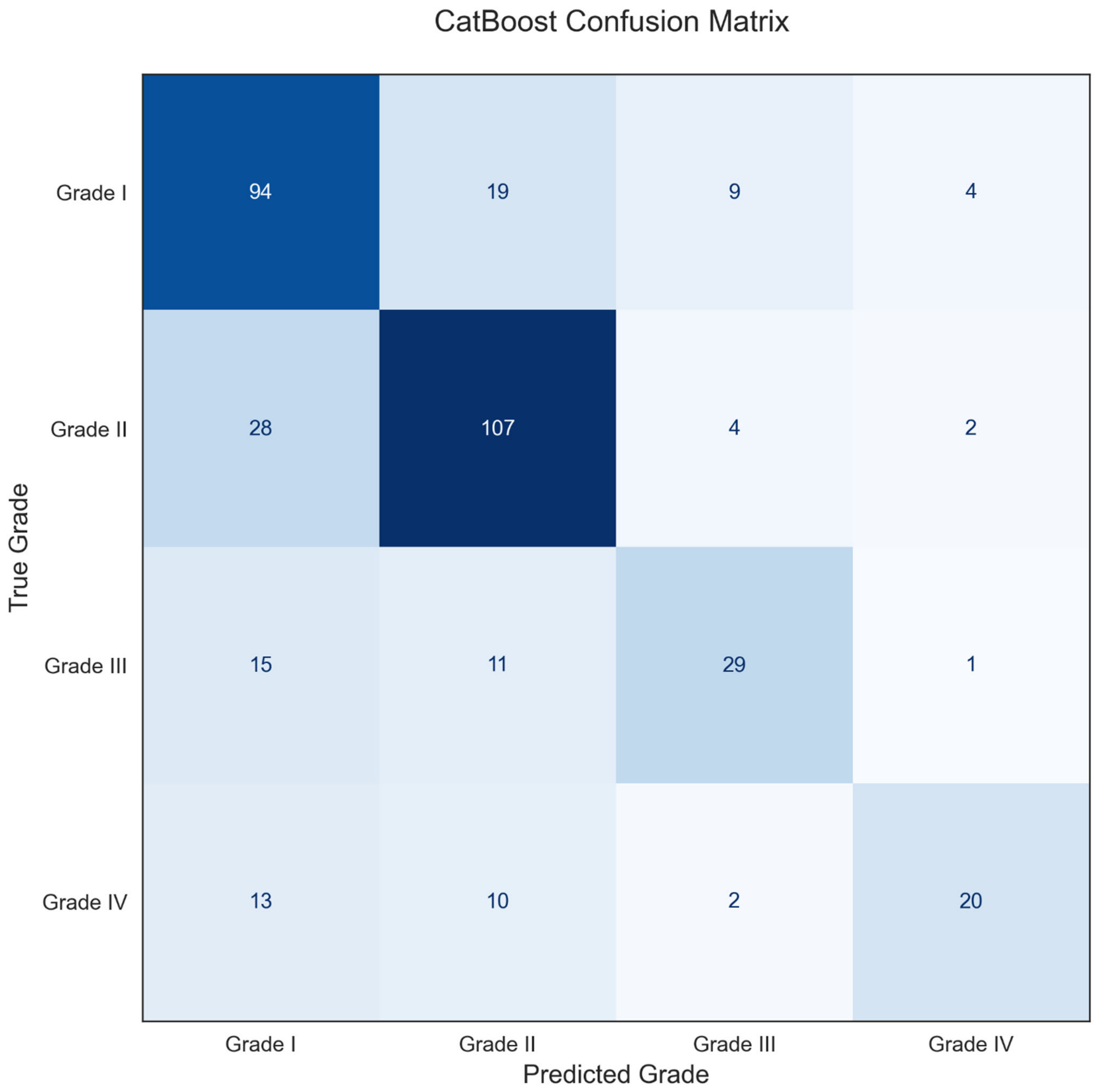

b) Logistic Regression

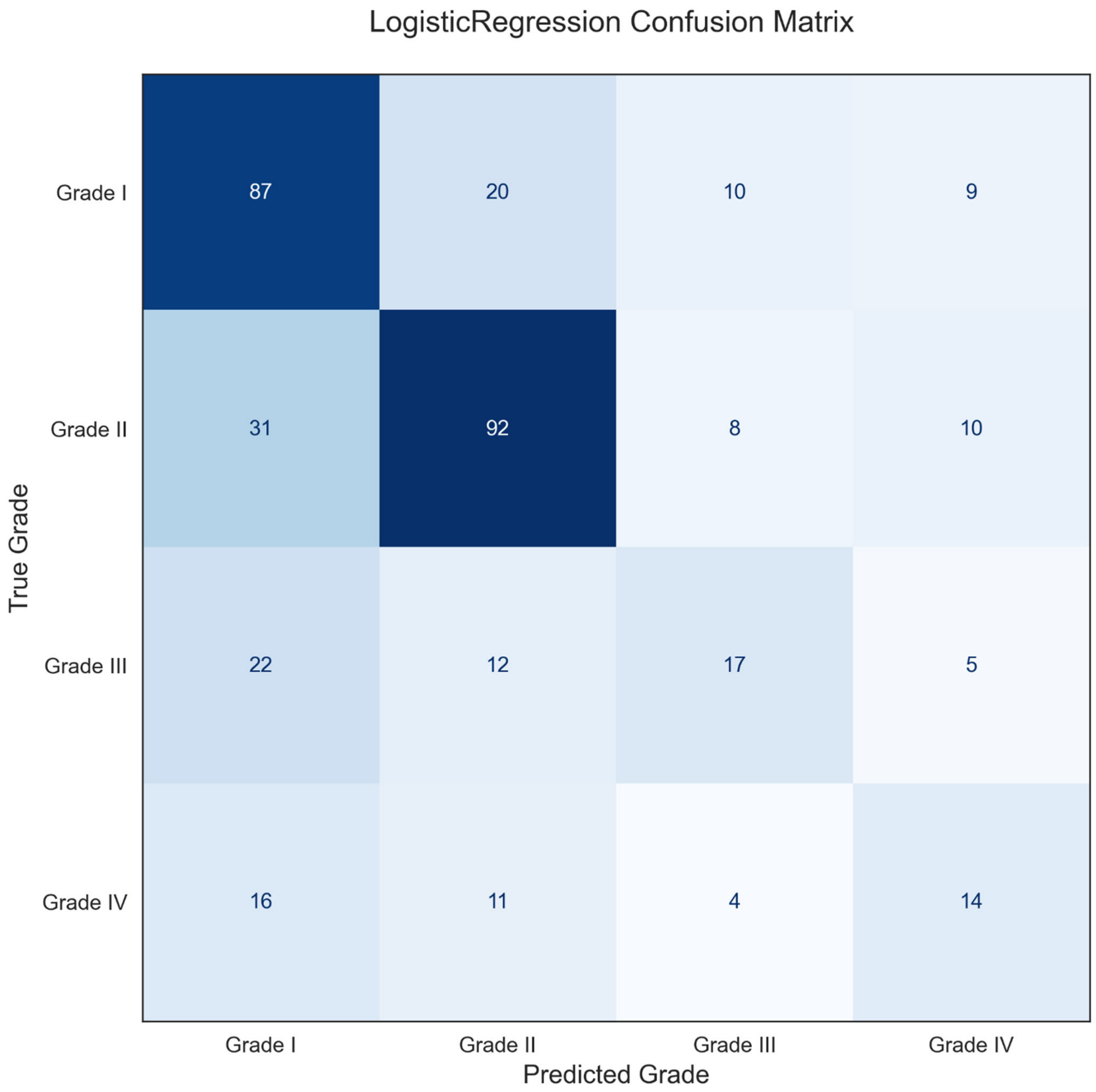

c) Random Forest

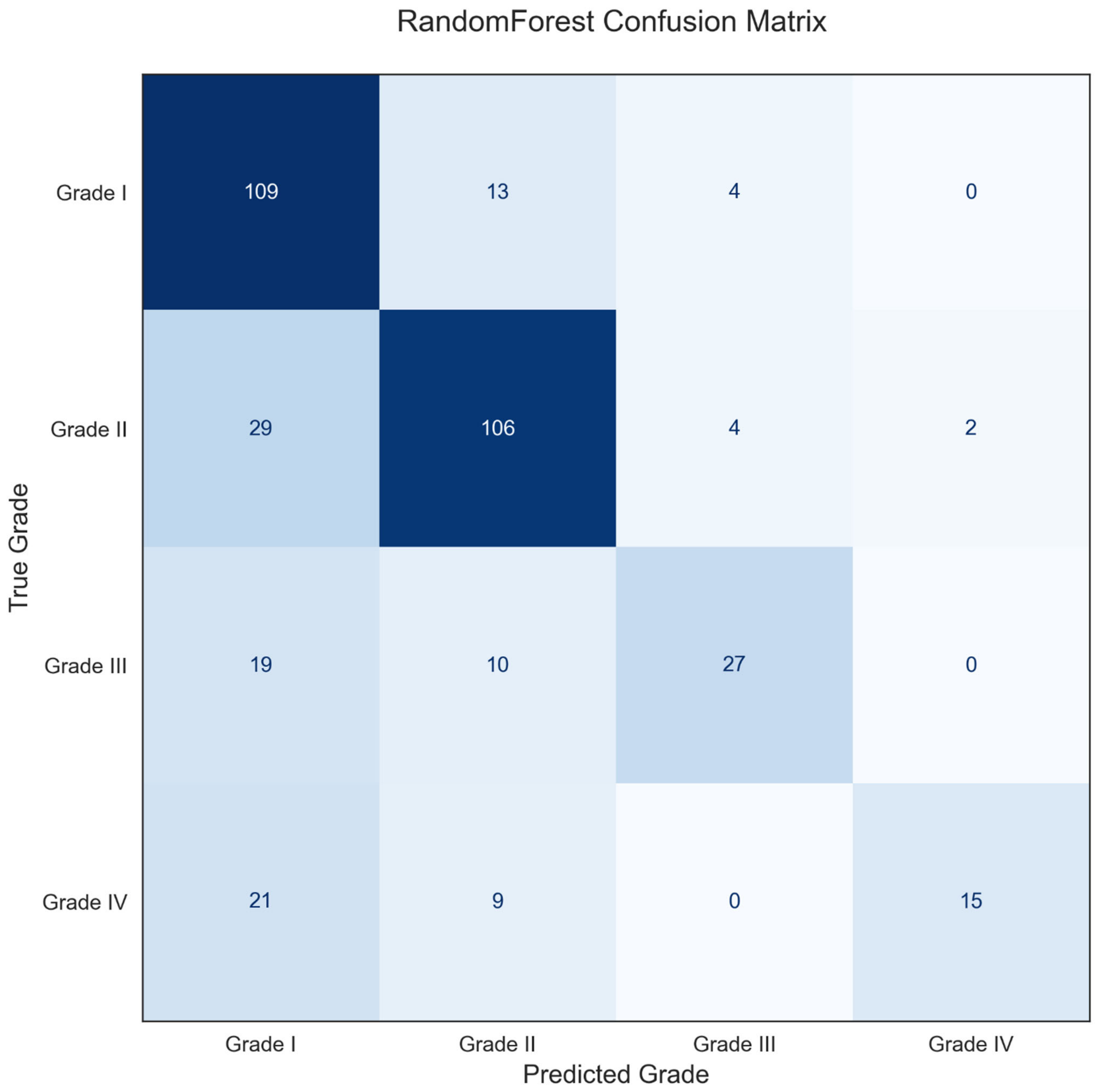

d) SVM

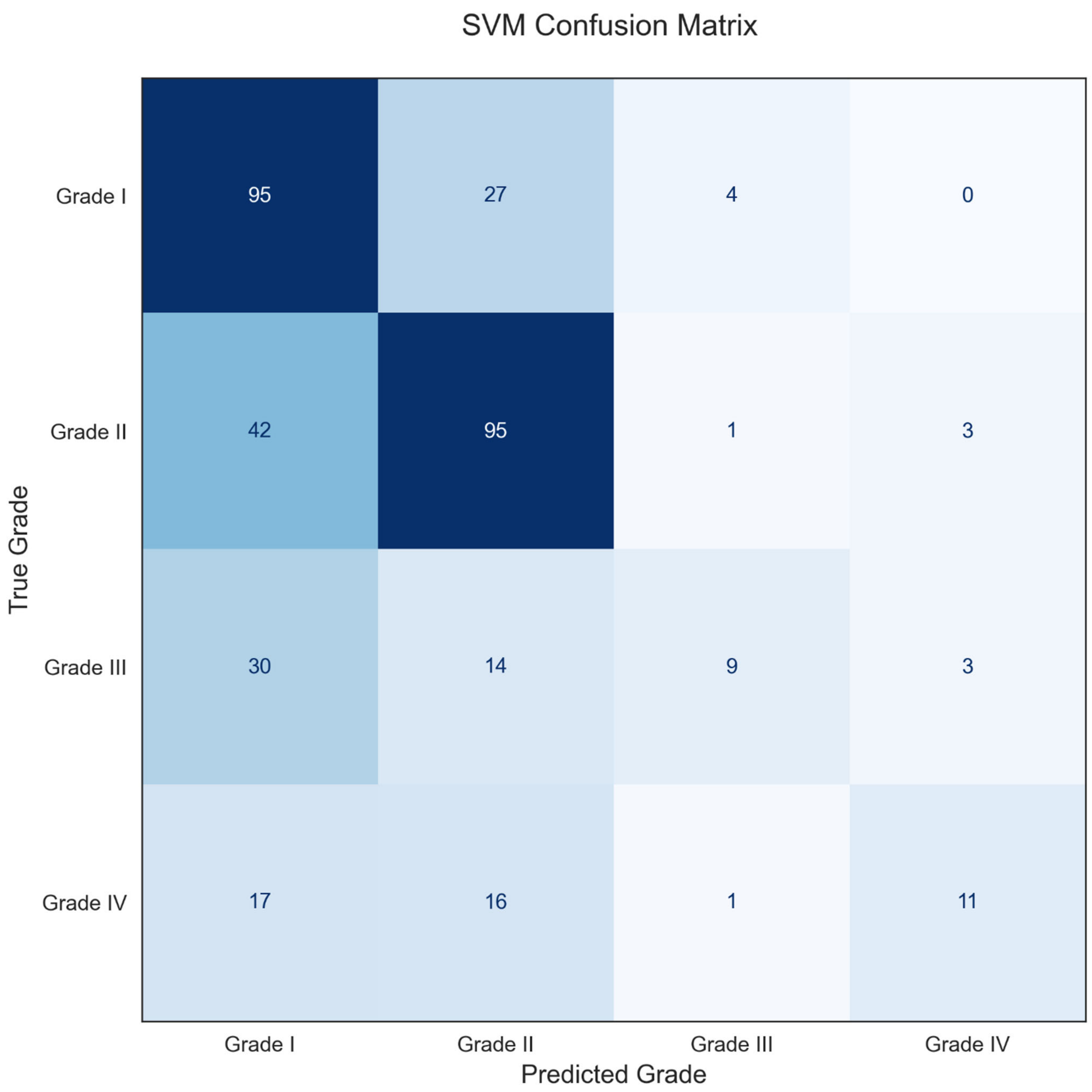
SVM Confusion Matrix
True Grade
Grade I
Grade II
Grade III
Grade IV
95
27
4
0
42
95
1
3
30
14
9
3
17
16
1
11
Grade I
Grade II
Grade III
Grade IV
Predicted Grade

e) XGBoost

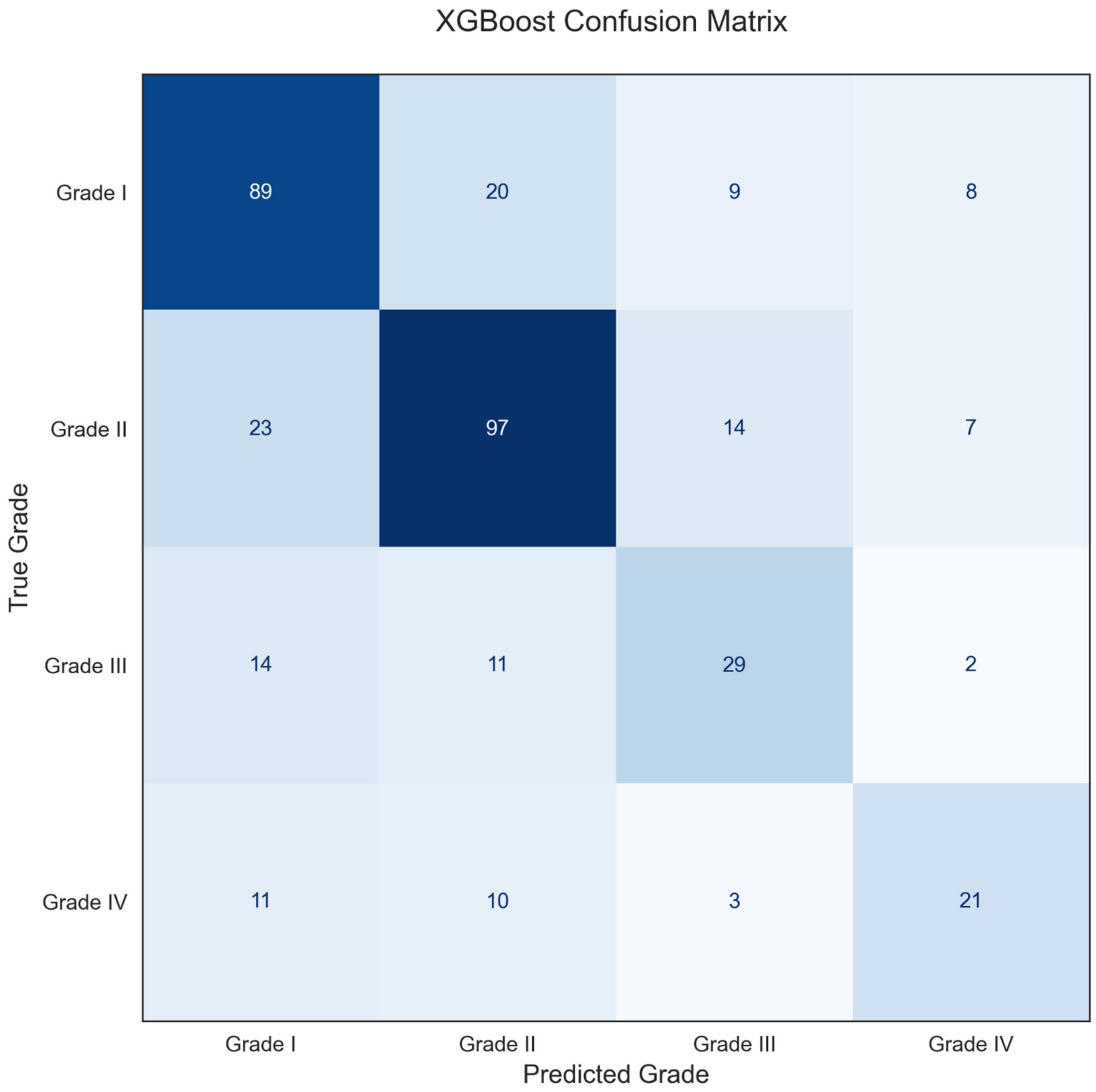

XGBoost Confusion Matrix
True Grade
Grade I
Grade II
Grade III
Grade IV
89
20
9
8
23
97
14
7
14
11
29
2
11
10
3
21
Grade I
Grade II
Grade III
Grade IV
Predicted Grade

Supplementary Figure S3. Feature Importance

a) CatBoost

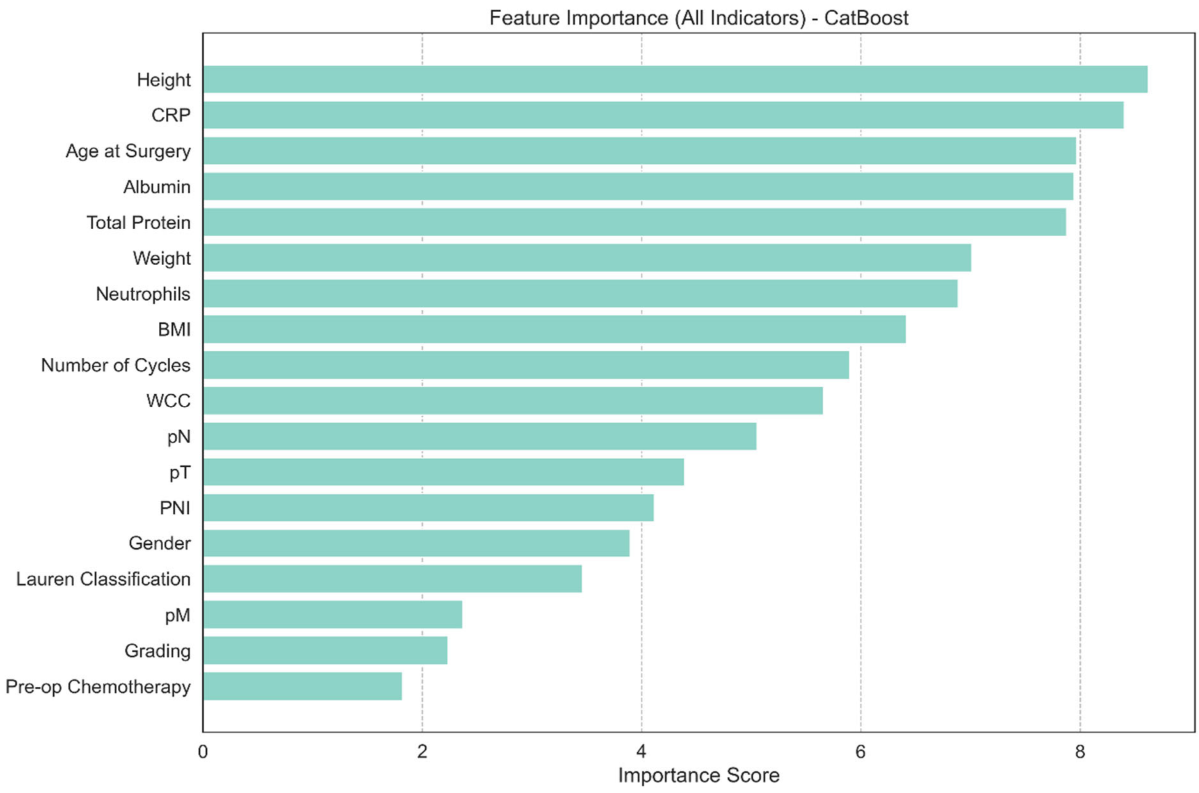

b) Random Forest

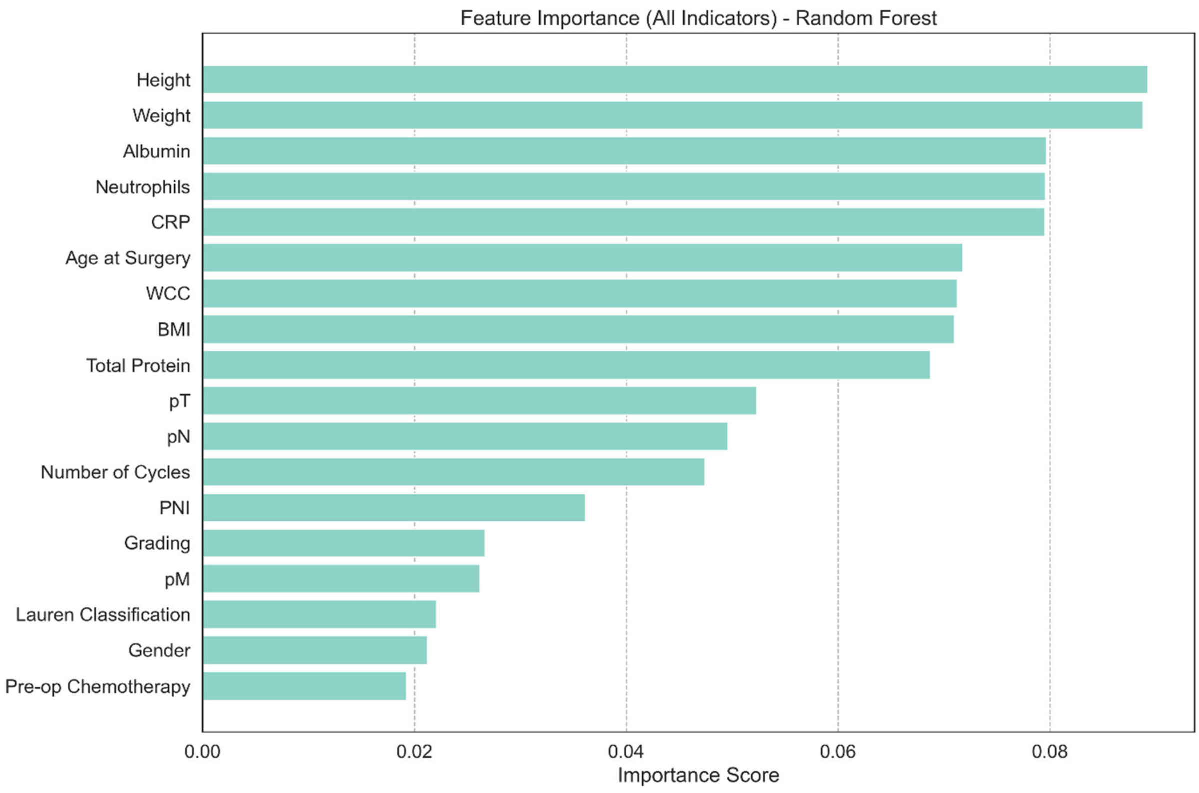

c) Logistic Regression

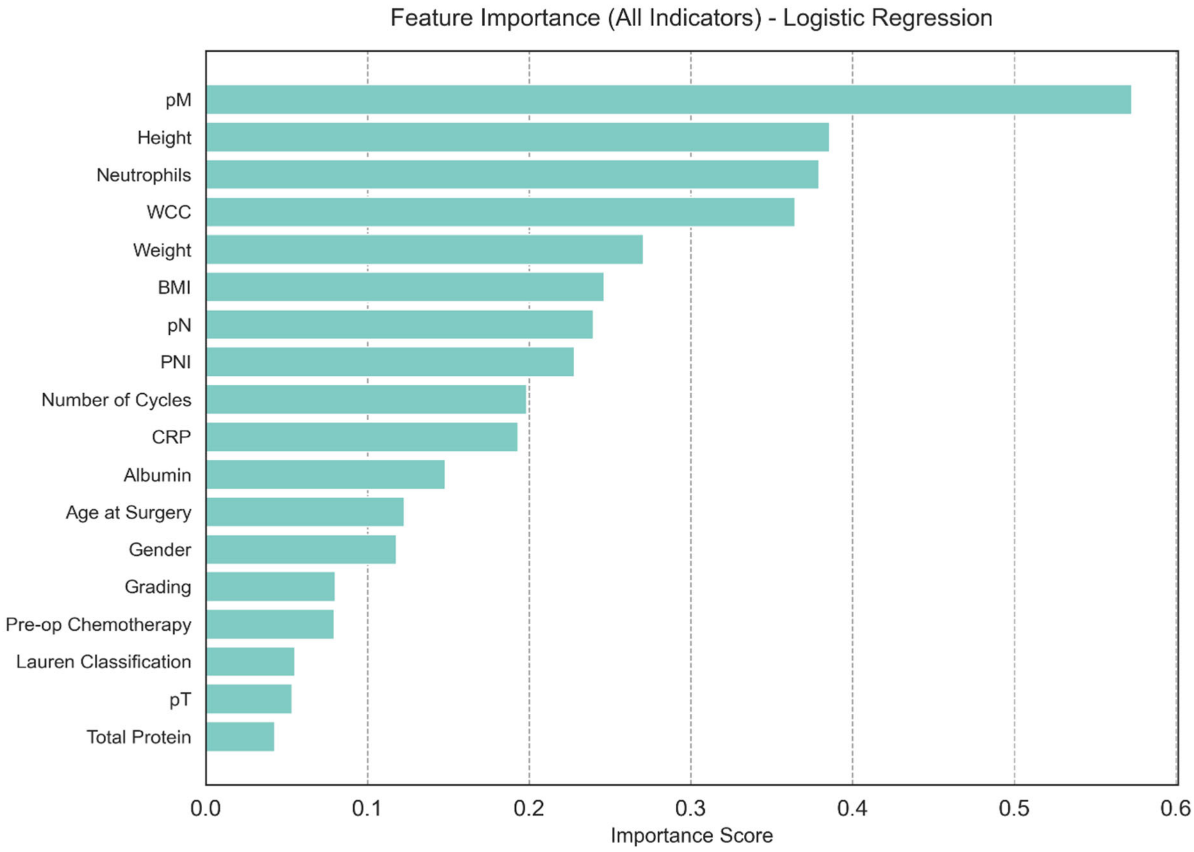

d) SVM

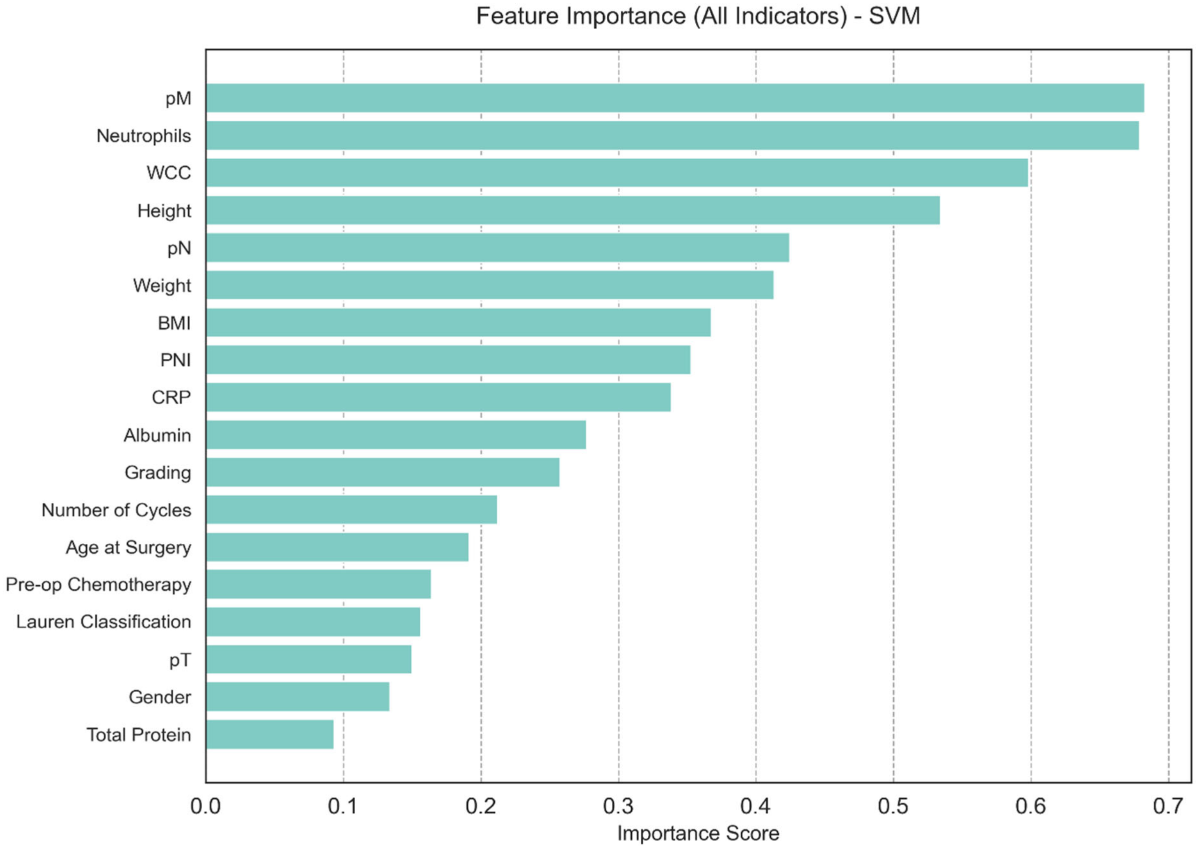

e) XGBoost

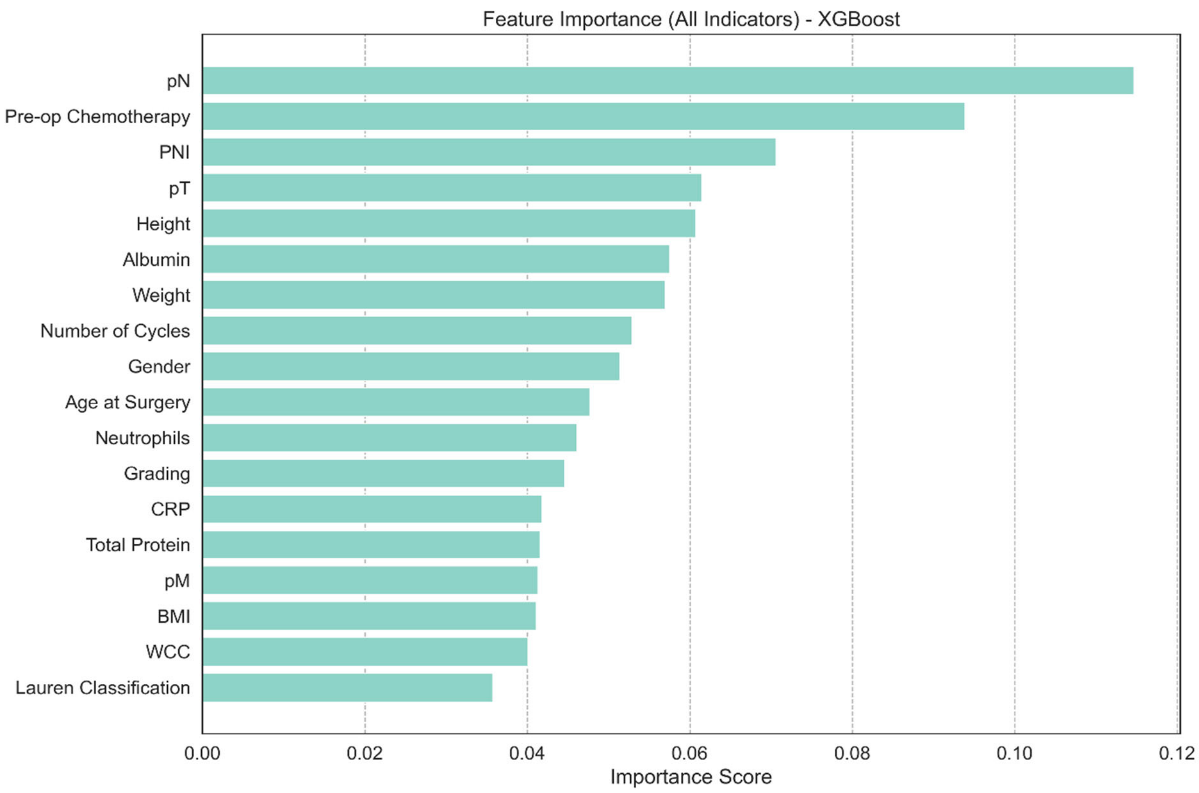

BMI – Body Mass Index; CRP – C-reactive Protein; Number of Cycles – Neoadjuvant Chemotherapy Cycles; PNI – Prognostic Nutritional Index; pN – pathological nodal status; pT – pathological tumor status; pM – pathological metastasis

Supplementary Figure S4. SHAP

a) Bar

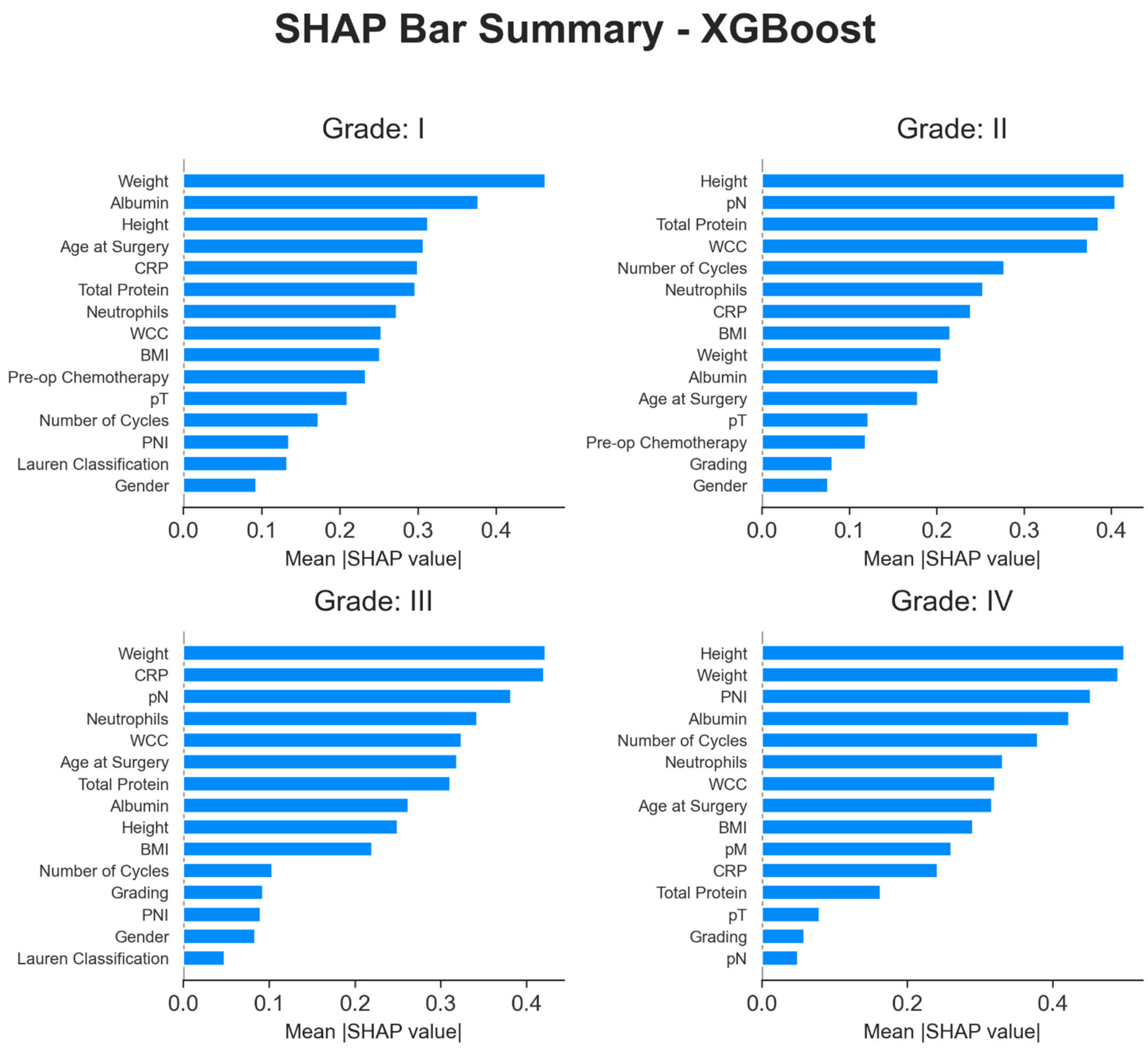

b) Beeswarm

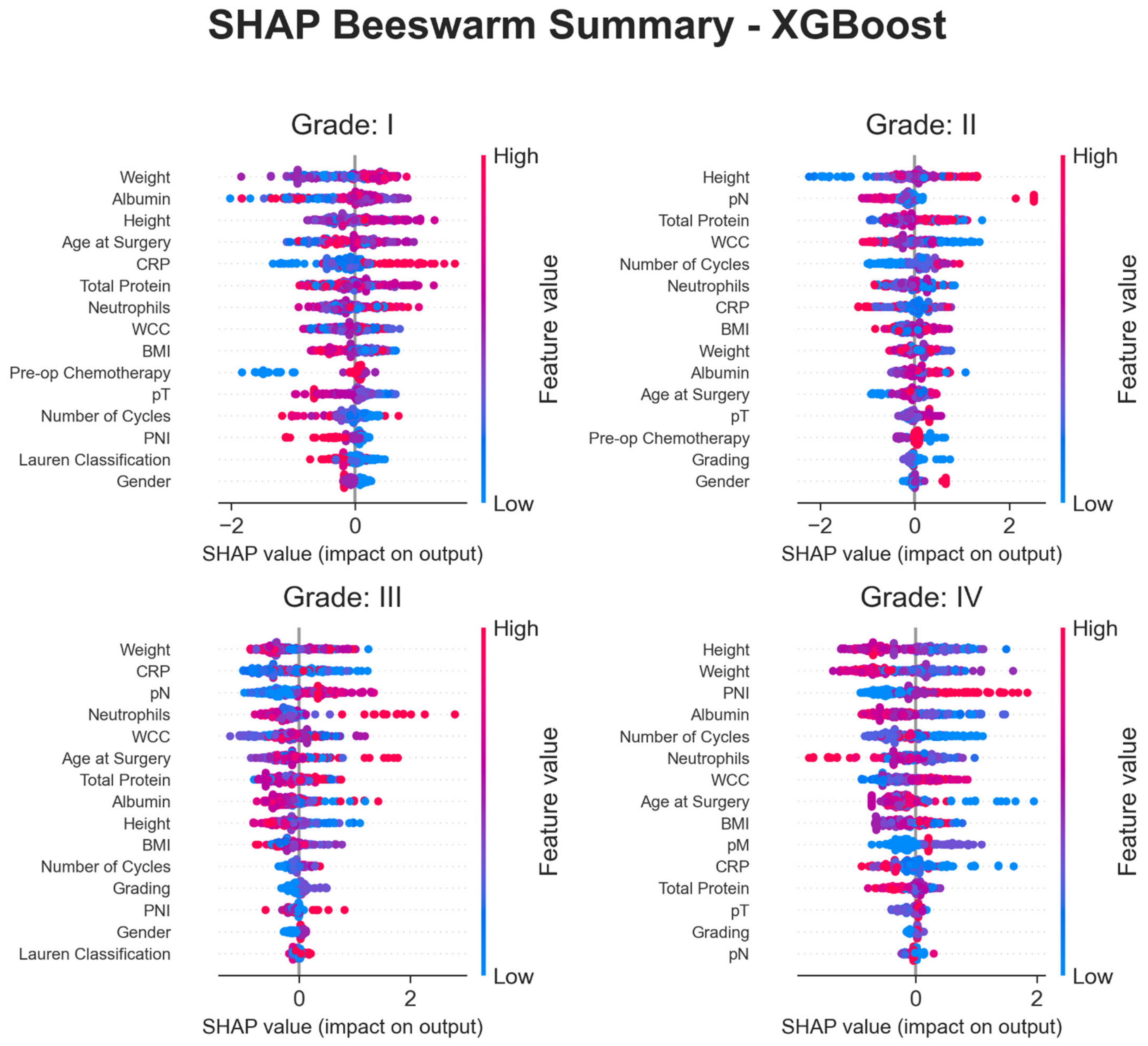
SHAP Beeswarm Summary - XGBoost
Grade: I
Weight
Albumin
Height
Age at Surgery
CRP
Total Protein
Neutrophils
WCC
BMI
Pre-op Chemotherapy
pT
Number of Cycles
PNI
Lauren Classification
Gender
−2
0
SHAP value (impact on output)
High
Low
Feature value
Grade: II
Height
pN
Total Protein
WCC
Number of Cycles
Neutrophils
CRP
BMI
Weight
Albumin
Age at Surgery
pT
Pre-op Chemotherapy
Grading
Gender
−2
0
2
SHAP value (impact on output)
High
Low
Feature value
Grade: III
Weight
CRP
pN
Neutrophils
WCC
Age at Surgery
Total Protein
Albumin
Height
BMI
Number of Cycles
Grading
PNI
Gender
Lauren Classification
0
2
SHAP value (impact on output)
High
Low
Feature value
Grade: IV
Height
Weight
PNI
Albumin
Number of Cycles
Neutrophils
WCC
Age at Surgery
BMI
pM
CRP
Total Protein
pT
Grading
pN
0
2
SHAP value (impact on output)
High
Low
Feature value
